# Population genetic variation and geographic distribution of suitable areas of *Coptis* species in China

**DOI:** 10.3389/fpls.2024.1341996

**Published:** 2024-03-19

**Authors:** Yujie Chi, Changli Liu, Wei Liu, Xufang Tian, Juan Hu, Bo Wang, Di Liu, Yifei Liu

**Affiliations:** ^1^ College of Pharmacy, Hubei University of Chinese Medicine, Wuhan, China; ^2^ Hubei Institute for Drug Control, Wuhan, China; ^3^ Hubei Key Laboratory of Chinese Medicine Resource and Chemistry, Hubei University of Chinese Medicine, Wuhan, China

**Keywords:** *Coptis*, genetic diversity, population structure, environment variables, distribution of suitable area

## Abstract

**Introduction:**

The rhizomes of *Coptis* plants have been used in traditional Chinese medicine over 2000 years. Due to increasing market demand, the overexploitation of wild populations, habitat degradation and indiscriminate artificial cultivation of *Coptis* species have severely damaged the native germplasms of species in China.

**Methods:**

Genome-wide simple-sequence repeat (SSR) markers were developed using the genomic data of *C. chinensis*. Population genetic diversity and structure of 345 *Coptis* accessions collected from 32 different populations were performed based on these SSRs. The distribution of suitable areas for three taxa in China was predicted and the effects of environmental variables on genetic diversity in relation to different population distributions were further analyzed.

**Results:**

22 primer pairs were selected as clear, stable, and polymorphic SSR markers. These had an average of 16.41 alleles and an average polymorphism information content (PIC) value of 0.664. In the neighbor-joining (N-J) clustering analysis, the 345 individuals clustered into three groups, with *C. chinensis*, *C. chinensis* var. *brevisepala* and *C. teeta* being clearly separated. All *C. chinensis* accessions were further divided into four subgroups in the population structure analysis. The predicted distributions of suitable areas and the environmental variables shaping these distributions varied considerably among the three species.

**Discussion:**

Overall, the amount of solar radiation, precipitation and altitude were the most important environmental variables influencing the distribution and genetic variation of three species. The findings will provide key information to guide the conservation of genetic resources and construction of a core reserve for species.

## 1 Introduction

The genus *Coptis* Salisb. of the family Ranunculaceae is important for medicinal herbs and widely used in healthcare (Wang et al., 2019). The rhizomes of *Coptis* species (Coptidis Rhizome, CR) have been used in traditional Chinese medicine for over 2000 years; the first records of their use in medicines appear in the Divine Farmer’s Materia Medica (Shennong Bencao Jing) during the Han dynasty (202 B.C.–220 A.D.) (Liu et al., 2021). According to the Chinese Pharmacopoeia, CR includes the dry rhizomes of *C. chinensis* Franch., *C. deltoidea* C.Y. Cheng et Hsiao, and *C. teeta* Wall., which are known as ‘Weilian’, ‘Yalian’ and ‘Yunlian’, respectively. Thus far, studies on *Coptis* plants have primarily focused on the chemical constitution and pharmacological effects of their main active ingredients, which include various protoberberine-type alkaloids such as berberine, coptisine, jatrorrhizine, palmatine, columbamine, and epiberberine (Lv et al., 2016; Yang et al., 2017). These alkaloids are known to have preventative and therapeutic effects on diabetes, cancer, cardiovascular, and nervous system diseases (Wu et al., 2016; Chou et al., 2017).

*Coptis* plants typically grow in cold, humid, and shady habitats in mountains or valleys at altitudes ranging from 1,200–2,000 m.s.l (Zhao et al., 2021). In China, *Coptis* species are distributed throughout the southern and southwestern mountain ranges, including the Himalayas (Wang et al., 2020). Owing to their narrow geographical distribution, the natural resources of *Coptis* are limited (Wang et al., 2022b). The market demand for CR has been increasing, especially in recent years (Ma et al., 2020; Xu et al., 2022). However, overexploitation and habitat destruction over the long-term have severely impacted wild *Coptis* populations and depleted the natural resources of these plants. At present, almost all CRs available on the market are derived from cultivated germplasms. Most of these CRs are from *C. chinensis*, with those of *C*. *deltoidea* and *C*. *teeta* being less common (Wang et al., 2022b). As a variety of *C. chinensis*, populations of *C. chinensis* var. *brevisepala* W.T. Wang et Hsiao are also in decline owing to the habitat degradation and overexploitation (Mamut et al., 2018). The molecular mechanisms modulating the signaling pathway of *Coptis* pathogenesis have been widely studied (Yamada et al., 2016; He et al., 2018; Chen et al., 2020b). A recent analysis of the *C*. *chinensis* genome found that the evolution of a Ranunculales clade-specific gene family, CYP719, greatly contributed to the diversification of protoberberine-type alkaloids in these plants (Liu et al., 2021). However, there is limited knowledge on the genetic relationships between various *Coptis* species as well as the genetic diversity associated with the adaptation of *Coptis* species across a range of environments.

The complex interplay between genes and environments shapes the population structures and phenotypes of plant species (Li et al., 2021). Changes in the environment can significantly impact the quality of medicinal herbs (Yang et al., 2018; Perrino et al., 2023). For instance, environmental change can influence the medicinal properties of medicinal herbs by affecting gene expression and consequently altering the abundances of secondary metabolites (Blanquart et al., 2013). At present, the rapid transferring and domestication of wild *Coptis* into cultivated germplasm as well as the indiscriminate propagation of *Coptis* are likely to negatively impact the rational layout of CR production and undermine the medicinal qualities of CR in the market (Han et al., 2019). The domestication history of *C. chinensis* is still largely unknown in the background of mixed germplasm. Whether it is single origin or multi origin domestication, as well as the hypothesis that the specific location of the original domestication center is in which province of Hubei, Sichuan, and Chongqing, the three main production areas of authentic medicinal herbs, has always been controversial. There were also hypothesis dividing cultivated *C. chinensis* into ‘Nananlian’ and ‘Beianlian’ based on the Yangtze River boundary (Liu et al., 2018). In addition, the impact of changes in environmental variables caused by different terrains on the domestication process of *C. chinensis* also needs to be explored. There is hence an urgent need to jointly evaluate the genetic variation and ecological suitability of *Coptis* species. Specifically, to mitigate additional losses of germplasm sources for *Coptis*, it is necessary to investigate the genetic diversity and architecture of current *Coptis* germplasms, as this will facilitate the selection and cultivation of superior *Coptis* germplasms in the future. As environmental conditions strongly influence the distributions of plant species, environmental variables can be employed to simulate plant niches (Poirazidis et al., 2019; Zhang et al., 2019). Knowledge of the distributions of ecologically suitable areas for *Coptis* species will also help in identifying the geographical and environmental factors influencing the contents of active ingredients in *Coptis* germplasm, thereby providing crucial data for the conservation and restoration of native *Coptis* populations.

Simple sequence repeats (SSRs) are effective and economical molecular markers for assessing the levels of genetic variation in plant populations and have been widely used for germplasm identification and breeding of both crops and medicinal herbs (Zhang et al., 2016; Lin et al., 2020; Chen et al., 2020a; Li et al., 2020a). In this study, genome-wide SSR markers for *C. chinensis* were developed, and 22 pairs of highly polymorphic primers were screened to analyze the genetic diversity and population structures of 345 wild and cultivated accessions derived from three *Coptis* species in China. Using 68 environmental variables, the potential regional distributions of ecologically suitable areas for the three *Coptis* taxa were predicted, and the environmental factors that significantly impacted their native distributions and genetic structure were identified. This study provided fundamental data and directions for germplasm conservation and breeding improvement of *Coptis* resources in future.

## 2 Materials and methods

### 2.1 Plant materials and DNA extraction

A total of 307 C. *chinensis* (including both cultivated and wild plants), 15 wild *C*. *teeta*, and 23 wild *C*. *chinensis* var. *brevisepala* were collected from 32 localities in China (**Supplementary Table 1**). Genomic DNA was extracted from the fresh or dried leaf tissues (using silica gel) using a Plant Genomic DNA Extraction Kit (BioTeke, Wuxi, China) in accordance with the manufacturer’s instructions. The quality and purity of the extracted DNA were evaluated using a NanoDrop 2000 spectrophotometer (Thermo Scientific, USA) and 1% agarose gel electrophoresis (Ouyang et al., 2018). Each DNA sample had a final concentration of approximately 50 ng/μL. The samples were subsequently used for PCR amplification (Liu et al., 2020).

### 2.2 Identification and experimental validation of SSRs

Based on *C. chinensis* genome data (Accession number: GCA_15680905.1) downloaded from the NCBI database (Liu et al., 2021), SSRs were searched using the MISA software (http://pgrc.ipk-gatersleben.de/misa/), and the parameters were set to as follows: mono-, di-, tri-, tetra-, penta-, and hexa-nucleotides with minimum repeat numbers of 10, 6, 5, 5, 5, and 5, respectively. Compound SSRs were identified as any two or more SSRs that had a maximum interruption of three bases. SSR primers were designed using Primer Premier 5.0 (http://www.premierbiosoft.com/). Given that dinucleotide and trinucleotide SSRs comprise the majority of highly polymorphic sites in plants (Wang et al., 2014), 180 pairs of dinucleotide and trinucleotide-based primers were generated. The M13 universal connector sequence (GTAAAACGACGGCCAGT) was added to the forward primers 5’ to supplement the generation of the reverse primers. The corresponding M13 connectors were modified by four different fluorescent groups: FAM (blue), HEX (green), ROX (red), and TAMRA (yellow).

The continually adjusted and optimized reaction system contained 2 µL genomic DNA, 5 µL 2 × Taq PCR MasterMix, 0.05 µL forward primer, 0.24 µL reverse primer, 0.15 µL M13 connector (FAM/HEX/ROX/TAMRA), and 2.56 µL ultrapure water. The mixed PCR amplifications were performed in a BiometraTone 96G PCR cycler (Analytik Jena AG, Jena, Germany) with a PCR amplification cycle of 94 °C pre-denaturation for 5 min, followed by 35 cycles of 94 °C for 30 s, 55 °C annealing for 30 s, 72 °C extension for 30 s, and a 72 °C extension for 10 min. The PCR products were then identified using an ABI 3730xl Genetic Analyzer Sequencer (Sangon Biotech Co., Ltd., Shanghai, China), the outputs were analyzed in the software GeneMarker v2.2.0 (SoftGenetics, State College, Pennsylvania, USA), and the peak information were recorded in diploid form.

### 2.3 Screening of species occurrence data and environmental variables

In addition to the 32 localities from which *Coptis* plants had been sampled, 204 occurrence points were identified from previously published articles as well as the public databases of the China National Knowledge Infrastructure (CNKI) (https://www.cnki.net), the China Digital Plant Herbarium (CVH) (https://www.cvh.ac.cn), and the Global Biodiversity Information Facility (GBIF) (https://www.gbif.org). Subsequently, MaxEnt modeling (Steven et al., 2006) was performed using datasets detailing the occurrences of 142 C. *chinensis*, 30 C. *teeta*, and 64 C. *chinensis* var. *brevisepala* (**Supplementary Table 2**).

*Coptis* plants prefer cold, humid, and semi shaded natural environments (Li et al., 2020b). Based on their growth habits, this study focuses on environmental variables such as altitude, temperature, light, rainfall, and water vapor pressure. A suite of bioclimatic variables were retrieved from the WorldClim database (https://www.worldclim.org). These included altitude, 19 ‘bioclimatic’ variables that were measured from 1970 to 2000 (BIO1: Annual Mean Temperature; BIO2: Mean Diurnal Range; BIO3: Isothermality; BIO4: Temperature Seasonality; BIO5: Max Temperature of Warmest Month; BIO6: Min Temperature of Coldest Month; BIO7: Temperature Annual Range; BIO8: Mean Temperature of Wettest Quarter; BIO9: Mean Temperature of Driest Quarter; BIO10: Mean Temperature of Warmest Quarter; BIO11: Mean Temperature of Coldest Quarter; BIO12: Annual Precipitation; BIO13: Precipitation of Wettest Month; BIO14: Precipitation of Driest Month; BIO15: Precipitation Seasonality; BIO16: Precipitation of Wettest Quarter; BIO17: Precipitation of Driest Quarter; BIO18: Precipitation of Warmest Quarter; BIO19: Precipitation of Coldest Quarter), as well as monthly measurements of precipitation (P1-12), solar radiation (SR1-12), water vapor pressure (WVP1-12) and temperature (T1-12). All variables had a spatial resolution of 2.5 arc-minutes. Using data on the occurrences of *Coptis* species and 68 environmental parameters, the statistical significance of each variable in predicting the occurrences of *Coptis* species was assessed using the Jackknife approach. Next, Pearson correlation coefficients were calculated between the 68 environmental variables, and statistically significant associations between environmental variables were identified based on a cutoff of |R| ≥ 0.80 (Shcheglovitova and Anderson, 2013). For each pair of substantially correlated environmental variables, only the variable with the larger contribution was retained in the model (Yang et al., 2021; Wei et al., 2021a).

### 2.4 Model reliability test and classification of suitable regions

Maximum entropy models for *C*. *chinensis*, *C*. *teeta*, and *C*. *chinensis* var. *brevisepala* were constructed using the MaxEnt v3.4.1 software (Phillips et al., 2017). To ensure that the distributions of the different *Coptis* species would approximate a normal probability distribution, 75% of the data was used for model training, while the remainder was used for model testing. To reduce the likelihood of model errors, the maximum number of parameters was set to 10,000, and each method was repeated ten times; all other parameters were set to default (Sun et al., 2020; Yan et al., 2020). The accuracies of model predictions were assessed using the area enclosed by the receiver operating characteristic curve (AUC), which had a range of (0, 1). Increasing AUC values indicate higher credibility in distinguishing between appropriate and inappropriate situations, with an AUC value of 0.9 indicating a very accurate model prediction (Guo et al., 2019; Liu et al., 2019).

The result of each MaxEnt model was converted into grid data and imported into ArcGIS 10.5. A reclassification process was then applied to identify ecologically suitable regions for a species. According to previous research results, the maximum test sensitivity plus specificity (MTSPS) criterion is preferable to other threshold choices for the classification of acceptable locations (Tang et al., 2018). Areas with suitability values below the threshold were declared unsuitable in line with the MTSPS criterion. Three equally sized sections of a species’ range of suitability between the MTSPS and 1 were chosen to represent the regions of low, moderate, and high suitability, respectively (Ye et al., 2018; Wei et al., 2021b).

### 2.5 Statistical analysis

Based on peak position data, GenALEx6.5 software (Peakall and Smouse, 2012) was used to calculate the following parameters of genetic diversity: the number of alleles (*N*_a_), the effective number of alleles (*N*_e_), the observed heterozygosity (*H*_o_), the expected heterozygosity (*H*_e_), the value of Shannon’s information index (*I*), the inbreeding coefficient (*F*_is_), the values between pairs indicative of population divergence (*F*_st_) and the gene flow between populations (*N*_m_). In addition, GenALEx6.5 was used for the analysis of molecular variance (AMOVA) and a Mantel correlation test between the Nei genetic distances and the geographical distances among separate populations. Polymorphism information content (*PIC*) was calculated using PowerMarker version 3.25 (Liu and Muse, 2005). To avoid the impact of a small sample size, 19 *Coptis* populations with a sample size greater than 5 were selected for the calculation of population genetic diversity related parameters. Adjusted genetic information data to “0/1” format, with peaks marked as “1” and no peaks marked as “0”. Based on the “0/1” dataset, Analysis of Phylogenetics and Evolution (APE) package (Paradis and Schliep, 2019) in R software was used to generate a neighbor-joining (N-J) tree of all 345 *Coptis* samples. A principal coordinates analysis (PCoA) was also performed based on the genetic distance matrix calculated by GenALEx6.5. Population structure was established using STRUCTURE version 2.3.4 (Pritchard et al., 2000). Subsequently, the optimal *K* value by the method of Evanno (Evanno et al., 2010) was identified by importing the compressed package of the result file into Structure Harvester (https://taylor0.biology.ucla.edu/structureHarvester/).

Geographical populations of *Coptis* species displaying distinct genetic structures were used in investigations of the relationship between environmental factors and the genetic structures of *Coptis* plants. The eight environmental factors with the largest contributions to the modeled distribution of each species were chosen. Data for environmental variables were retrieved for each occurrence point using ArcGIS 10.5. The Wekemo Biocloud cloud service (https://bioincloud.tech) was then employed to perform a redundancy analysis (RDA) or a canonical correspondence analysis (CCA) based on the gradient length calculated by a trend correspondence analysis (DCA). To explore the driving effects of high contribution environmental variables on the genetic variation of *Coptis* species and cultivated *C*. *chinensis*, six groups (Group I, II, III-1, III-2, III-3 and III-4) and three subgroups (Group III-2, III-3 and III-4) were analyzed, respectively. Specifically, RDA was used when gradient length < 3.0, CCA was used when gradient length > 4.0, and both methods were used for any gradient length that ranged from 3.0 – 4.0.

## 3 Result

### 3.1 Identification and characterization of SSRs

A total of 340,387 SSRs belonging to six classes of microsatellites were detected in the *C. chinensis* genome, with an average of one SSR per 217.5 bp (**Supplementary Tables 3**, **4**). Of these SSRs, 48,923 were compound SSR loci with motifs that contained two types of repeats (14.37%). The most abundant type of repeat motif was mononucleotide (238,931, 70.20%), followed by dinucleotide (66,049, 19.4%) and trinucleotide (29,001, 8.52%) (**Supplementary Tables 3**, **4**). The repeat number of different SSR motif types varied considerably, from 5 (5.59%) to 12 (6.92%). The three most frequently occurring SSR types had major repeats of 10, 6 and 5 for mononucleotides, dinucleotides, and trinucleotides, respectively (**Supplementary Table 5**). In addition, the A/T repeat was dominant in mononucleotides (95.16%). In comparison, the AT/AT repeat was more prevalent in dinucleotides (57.18%), while the AAT/ATT repeat type accounted for 44.17% of trinucleotides (**Supplementary Table 6**).

Primer pairs from 180 randomly selected SSRs were designed and tested for their ability to detect polymorphisms in the *Coptis* samples. Of these, 22 SSR primer pairs showed high amplification efficiency, good reproducibility, and high polymorphism between samples, while 72 SSRs failed to amplify any product. The 22 SSRs with high polymorphism were thus used for further genetic analysis (**Supplementary Table 7**). These SSRs resulted in a total of 361 alleles, with the observed number of alleles (*N*_a_) varying from 6 (Ccssr49) to 31 (Ccssr3), and an average of 16.41 alleles per locus (**Supplementary Table 8**). The Ccssr3 locus was associated with the highest level of genetic variation, followed by the Ccssr53 locus. These loci showed high recognition efficiency and will be useful markers for distinguishing closely related *Coptis* species.

### 3.2 Genetic diversity and differentiation

**Figure 1:**
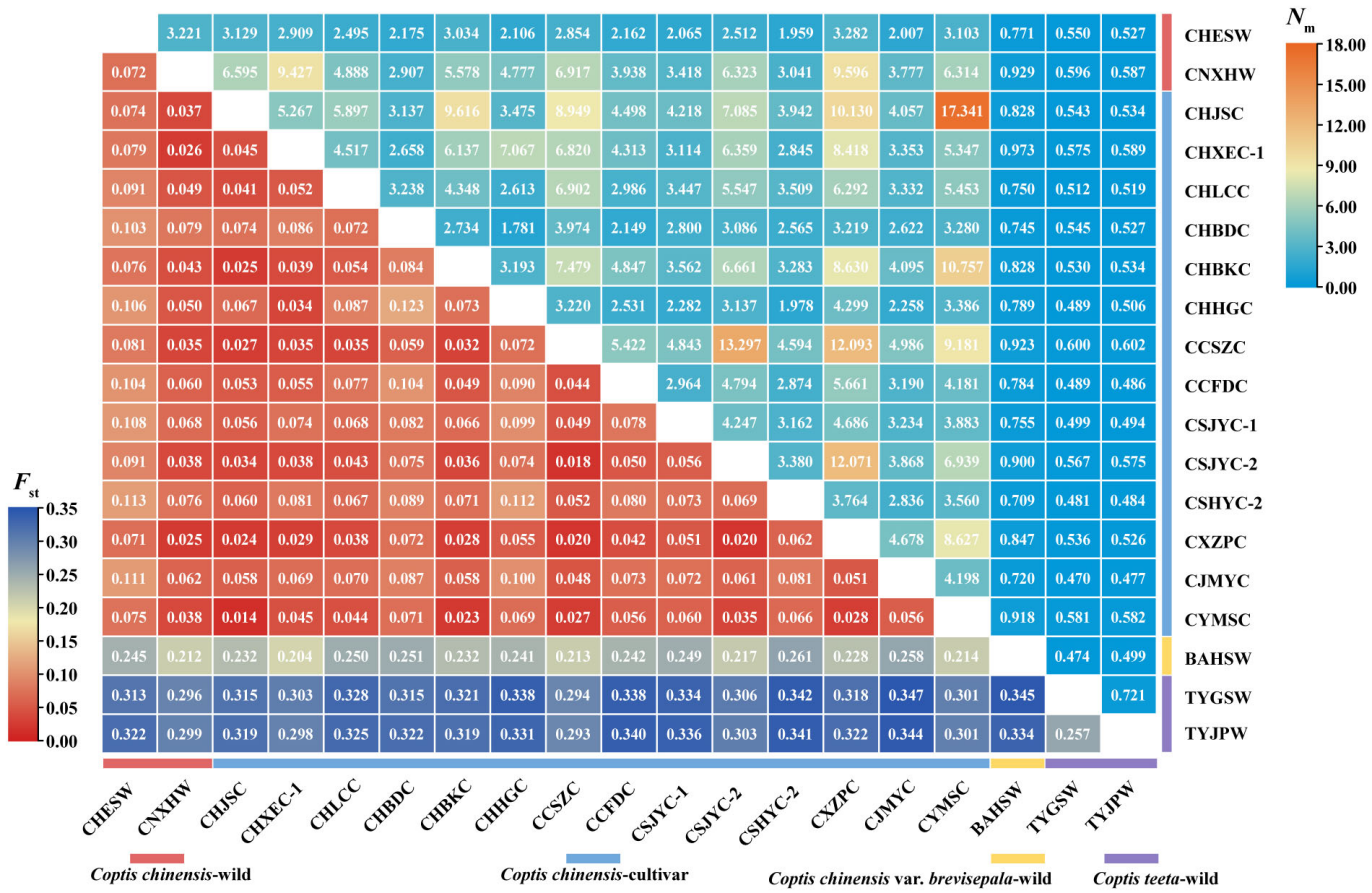
Heatmap of pairwise *F*_st_ and *N*_m_ values between different geographically *Coptis* populations. Numbers in the square were the pairwise *F*_st_ and *N*_m_ values between the populations in horizontal and vertical correspondingly. Colored ranges indicated different germplasm types.

The genetic diversity and differentiation of 19 *Coptis* geographical populations were estimated (**Figure 1**; **Table 1**). The average genetic diversity in *C. chinensis* populations was higher than that in *C. teeta* populations and *C. chinensis* var. *brevisepala* populations, as indicated by both the expected heterozygosity (*H*_e_) and Shannon’s information index (*I*). However, wild and cultivated *C. chinensis* populations did not differ significantly in their levels of genetic diversity. Deviating from expectation of the Hardey-Weinberg principle, the observed heterozygosity (*H*_o_) of the two *C. teeta* populations TYGSW and TYJPW was considerably higher than expected, while that of the *C. chinensis* var. *brevisepala* population BAHSW was considerably lower than expected. This research also compared the level of genetic diversity in samples of cultivated *C. chinensis* collected from three provinces: Hubei (HB), Sichuan (SC), and Chongqing (CQ). The results showed that the level of genetic diversity in cultivated accessions from HB (*I* = 1.475, n = 103) was slightly higher than that from SC (*I* = 1.371, n = 43) and CQ (*I* = 1.334, n = 43); this was in line with their differences in sample size (**Table 2**).

**Table 1:** Geographical origin and genetic diversity of 19 *Coptis* populations with sample size more than five.

Population ID	Origin	Latitude	Longitude	Altitude	Sample size	*N*_a_	*N*_e_	*I*	*H*_o_	*H*_e_	*PIC*	*F*_is_
CHESW	Enshi, Enshi, Hubei	30°26′29.82″	109°14′09.19″	1672	46	4.955	2.576	1.037	0.591	0.550	0.493	-0.039
CNXHW	Xinhuang, Huaihua, Hunan	27°19'27.10"	109°26'52.30"	1109	11	5.045	3.082	1.207	0.495	0.596	0.560	0.140
CHJSC	Jianshi, Enshi, Hubei	30°35′20.17″	109°34′20.25″	968	22	6.364	3.025	1.185	0.507	0.558	0.520	0.052
CHXEC-1	Xuanen, Enshi, Hubei	30°03′47.88″	109°49′21.77″	1759	19	6.864	3.621	1.346	0.594	0.616	0.585	0.002
CHLCC	Lichuan, Enshi, Hubei	30°23′18.47″	108°36′48.30″	1426	9	4.182	2.781	1.042	0.536	0.547	0.494	-0.003
CHBDC	Badong, Enshi, Hubei	30°50′52.62″	110°17′57.19″	1091	6	3.773	2.674	1.038	0.621	0.560	0.550	-0.135
CHBKC	Baokang, Xiangyang, Hubei	31°34′22.30″	111°06′22.80″	1212	24	6.091	3.027	1.200	0.497	0.569	0.533	0.102
CHHGC	Huanggang, Hubei	30°26′24.00″	114°52′12.00″	19	9	4.909	2.990	1.116	0.503	0.537	0.506	0.086
CCSZC	Shizhu, Chongqing	29°50'40.92"	108°14'10.46"	1350	30	6.227	2.948	1.246	0.622	0.608	0.564	-0.032
CCFDC	Fengdu, Chongqing	29°43′15.00″	107°52′18.00″	1350	6	3.682	2.599	0.976	0.439	0.518	0.474	0.152
CSJYC-1	Jiangyou, Mianyang, Sichuan	31°59'48.70"	104°45'48.02"	1260	12	4.227	2.596	1.030	0.573	0.539	0.536	-0.026
CSJYC-2	Jiangyou, Mianyang, Sichuan	31°42'08.00"	104°48'08.42"	620	12	5.318	2.967	1.210	0.627	0.598	0.555	-0.034
CSHYC-2	Hongya, Meishan, Sichuan	29°35'20.83"	103°16'28.02"	1270	6	3.091	2.327	0.875	0.545	0.497	0.485	-0.068
CXZPC	Zhenping, Ankang, Shaanxi	31°47′47.40″	109°24′37.76″	1674	24	6.727	2.978	1.212	0.569	0.558	0.525	-0.031
CJMYC	Muyang, Suqian, Jiangsu	34°14'33.40"	118°41'45.96"	108	12	4.591	2.586	1.025	0.560	0.519	0.520	-0.101
CYMSC	Mangshi, Yunnan	24°31′16.00″	98°31′47.00″	1512	20	6.364	3.254	1.284	0.525	0.603	0.566	0.127
BAHSW	Huangshan, Anhui	30°09′33.76″	118°07′42.05″	1264	23	5.364	3.300	1.133	0.361	0.542	0.504	0.366
TYGSW	Gongshan, Nujiang, Yunnan	27°33′43.00″	98°42′54.00″	1578	7	2.545	1.907	0.670	0.643	0.408	0.344	-0.549
TYJPW	Jinping, Honghe, Yunnan	22°46′50.27″	103°13′34.03″	1376	8	2.545	2.000	0.718	0.586	0.442	0.371	-0.291

**Table 2:** Genetic diversity of the cultivated *C. chinensis* samples in three provinces of Hubei (HB), Sichuan (SC) and Chongqing (CQ).

Population ID	Sample size	*N*_a_	*N*_e_	*I*	*H*_o_	*H*_e_	*PIC*	*F*_is_
HB	103	10.909	3.864	1.475	0.535	0.626	0.599	0.133
CQ	43	7.727	3.256	1.334	0.594	0.615	0.578	0.031
SC	43	8.045	3.438	1.371	0.583	0.623	0.586	0.070
mean	63	8.894	3.519	1.393	0.571	0.621	0.588	0.078

N_a_, number of alleles; N_e_, effective number of alleles; I, Shannon's information index; H_o_, observed heterozygosity; H_e_, expected heterozygosity; PIC, polymorphism information content; F_is_, inbreeding coefficient.

Very weak genetic differentiation was observed between the cultivated populations of *C. chinensis* (average pairwise *F*_st_ = 0.058), while that between two wild populations of the species (CHESW and CNXHW) was slightly higher (*F*_st_ = 0.072). In addition, CNXHW was found to be more genetically related to the cultivated populations of *C. chinensis* (average pairwise *F*_st_ = 0.049) than CHESW (average pairwise *F*_st_ = 0.092). A high level of genetic differentiation was observed between the two *C. teeta* populations, TYGSW and TYJPW (*F*_st_ = 0.257) (**Figure 1**). Importantly, the *C. chinensis* var. *brevisepala* population BAHSW showed a high level of genetic differentiation from the *C. chinensis* (average pairwise *F*_st_ = 0.234) and *C. teeta* populations (average pairwise *F*_st_ = 0.340). The level of genetic differentiation between the *C. chinensis* and *C. teeta* populations was also high (average pairwise *F*_st_ = 0.340) (**Figure 1**). The pairwise *N*_m_ value ranged from 0.470 to 17.341. A high level of gene flow was observed between cultivated *C. chinensis* populations, such as that between CHJSC and CYMSC (*N*_m_ = 17.341) and between CCSZC and CSJYC-2 (*N*_m_ = 13.297) (**Figure 1**). In particular, a very high level of gene flow (*N*_m_ > 12) was observed between CCSZC, CSJYC-2, and CXZPC, suggesting these populations were genetically related (**Figure 1**). The AMOVA analysis further showed that most genetic variation occurred within populations of the respective *Coptis* species. Nonetheless, relatively high levels of genetic variation among populations of *C. teeta* were also observed (**Supplementary Table 9**). A Mantel test showed a significant positive relationship (*R* ^2 ^ = 0.2072, *P* = 0.030) between the genetic and geographical distances of all 19 *Coptis* populations. However, when the Mantel test was conducted for the *C. chinensis* populations only, there was no correlation between their genetic and geographical distances (*R* ^2^ = -6.3777, *P* = 0.420) (**Supplementary Figure 1**).

### 3.3 Genetic relationship and structure of *Coptis* accessions

**Figure 2:**
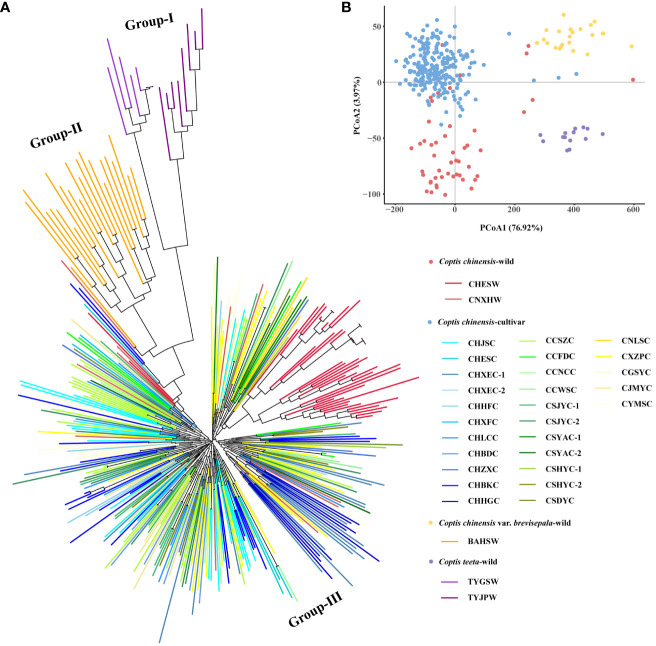
N-J tree **(A)** and PCoA **(B)** analysis of 345 accessions. Colored ranges indicated different germplasm types.

An N-J tree grouped all 345 *Coptis* accessions into three major groups (I, II and III) (**Figure 2A**). The wild populations of *C*. *teeta* and *C*. *chinensis* var. *brevisepala* formed their own groups, namely Group I and Group II, respectively. All *C*. *chinensis* individuals were clustered into the larger group, Group III. Within Group III, the wild *C. chinensis* population CHESW from Hubei was relatively independent, while most individuals of the wild *C. chinensis* population from Hunan (CNXEW) were mixed with cultivated accessions. These findings were consistent with the lower *F*_st_ and higher *N*_m_ values observed between CNXEW and the cultivated populations (**Figure 1**). The results of a PCoA analysis further supported the results of the N-J tree (**Figure 2B**).

**Figure 3:**
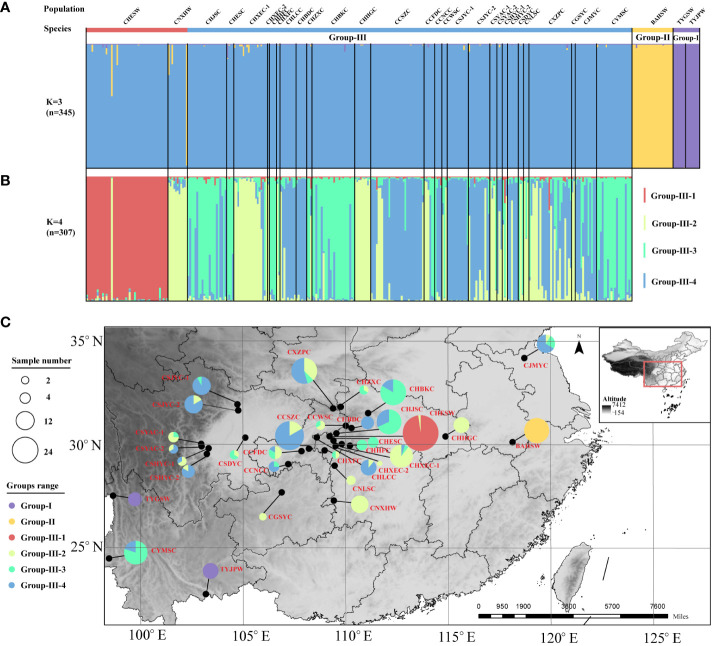
STRUCTURE analysis based on the entire SSR dataset. **(A)** The population structure of *Coptis* accessions (n = 345) when K = 3. **(B)** The population structure of *C. chinensis* accessions (n = 307) when *K* = 4. **(C)** The geographic distribution of the 32 *Coptis* populations inferred with STRUCTURE analysis. The different colors of the pie charts represented the proportions of the populations in the 6 Groups. Different circle sizes represented sample sizes.

In line with the result of the cluster analysis, the STRUCTURE analysis showed that the best population subdivision for all *Coptis* accessions was achieved when *K* = 3, corresponding to the three taxa well (**Figure 3A**). For both wild and cultivated *C. chinensis* samples in Group III (n = 307), further structural analysis revealed that the most suitable number of subgroups was *K* = 4 (**Figure 3B**). Almost all accessions from the wild *C*. *chinensis* population in Hubei (CHESW) belonged to Group III-1. The genetic structures of *C*. *chinensis* accessions from Sichuan and Chongqing, which were almost unanimous, constituted Group III-4. The wild and cultivated accessions from Hunan constituted Group III-2. In comparison, the cultivated *C. chinensis* populations from Hubei constituted a genetic admixture, with similar proportions of samples from Groups III-2, 3 and 4 (**Figure 3C**). This was consistent with the high level of genetic diversity documented in cultivated *C. chinensis* populations in Hubei (*I* = 1.475).

### 3.4 Potentially distribution of ecologically suitable areas and functional environment variables

**Figure 4:**
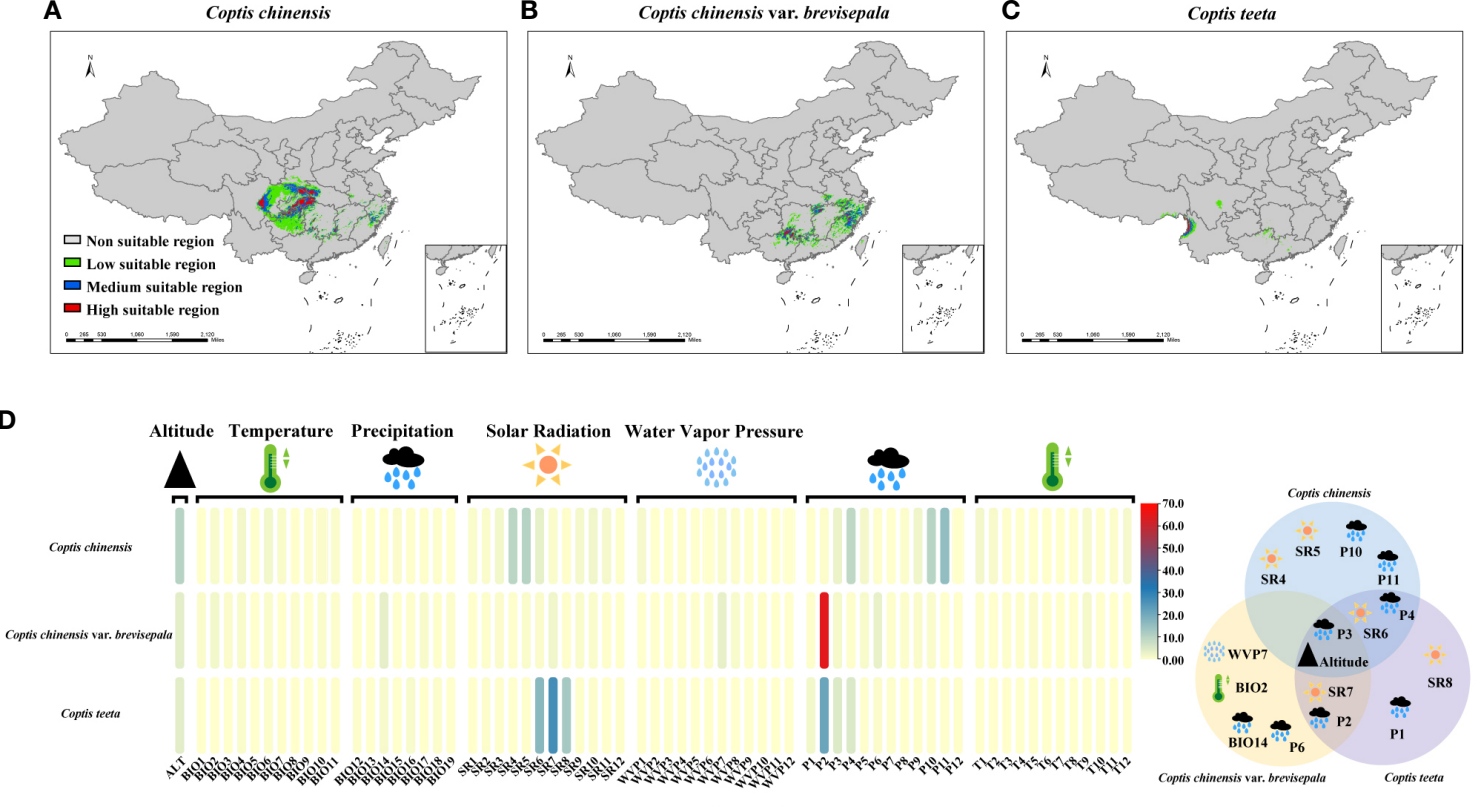
Analysis of ecological suitability of three *Coptis* species. The colored ranges indicated different species and suitable areas at different levels. The potential suitable distribution of *C. chinensis* **(A)**, *C.* *chinensis* var. *brevisepala* **(B)** and *C. teeta* **(C)** in China. **(D)** Contribution of 68 environmental variables to the distribution of three *Coptis* species. **(E)** Venn diagram of 16 contributing environmental variables affecting the distribution of three *Coptis* species. Effects of environmental variables classified into five categories (temperature, precipitation, solar radiation, water vapor pressure and altitude) on the distribution of *Coptis* species.

Under current climatic conditions, the distributions of suitable areas for the three *Coptis* species show little overlap (**Figure 4**). Areas that are predicted to be suitable for *C. chinensis* primarily occur in Hubei, Sichuan, Shaanxi, Hunan, Guizhou and Chongqing. Among these, highly suitable areas for *C. chinensis* are concentrated in two regions. The first region corresponds to the intersection of western Hubei, eastern Chongqing, northwestern Hunan, southern Shaanxi and northeastern Guizhou, while the second region is eastern Sichuan (**Figure 4A**). In comparison, the areas that are predicted to be suitable for *C. teeta* and *C. chinensis* var. *brevisepala* are considerably smaller and independent from those that are suitable for *C. chinensis* (**Figures 4B, C**). The distribution of suitable areas for *C. chinensis* var. *brevisepala* is relatively scattered, with highly suitable areas sporadically distributed across the provinces of Guizhou, Guangxi, Hubei, Hunan, Anhui, Zhejiang and Fujian (**Figure 4B**). Areas suitable for *C. teeta* are mainly distributed in Yunnan and eastern Tibet, and highly suitable areas of this species are restricted to the Gaoligong Mountains (**Figure 4C**).

To investigate environmental factors influencing the distributions of the three *Coptis* taxa, 68 environmental variables indicative of temperature (BIO1-11, T1-12), precipitation (BIO12-19, P1-12), solar radiation (SR1-12), water vapor pressure (WVP1-12) and altitude were analyzed. In general, variables relating to solar radiation and precipitation were found to be the main factors determining the current distribution of *Coptis* species; these were followed by variables relating to altitude and temperature (**Figure 4D**; **Supplementary Table 10**). In terms of seasonality, the most important variable affecting the distribution of *C. chinensis* in spring and early summer was the amount of solar radiation (25.2% in March, April, May, and June), while that for spring and autumn was precipitation (13.4% in March, April, and May and 28% in September, October, and November). Likewise, the dominant variable determining the distribution of *C. teeta* in summer was solar radiation (56.3% in June, July, and August), while that for late winter and early spring was precipitation (33% in December, January, February, and March). In comparison, the distribution of *C. chinensis* var. *brevisepala* was found to be influenced most by the level of precipitation in February, which accounted for 64.6% of variation in the species’ distribution.

### 3.5 Environmental variables related to population structure

Based on the results of the STRUCTURE analysis, 22 geographical populations (in each population ≥ 75% samples had a similar genetic background) of *Coptis* species belonging to six distinct genetic groups were selected for a study on the relationship between environmental and genetic variation in *Coptis* plants. Sixteen environmental variables, corresponding to the top eight environmental variables influencing the distribution of each of the three *Coptis* species, were selected for the study (**Figure 4E**). Of these, six variables had a large effect on the distribution of at least two species, while altitude and the level of precipitation in March significantly affected the distributions of all three *Coptis* species.

**Figure 5:**
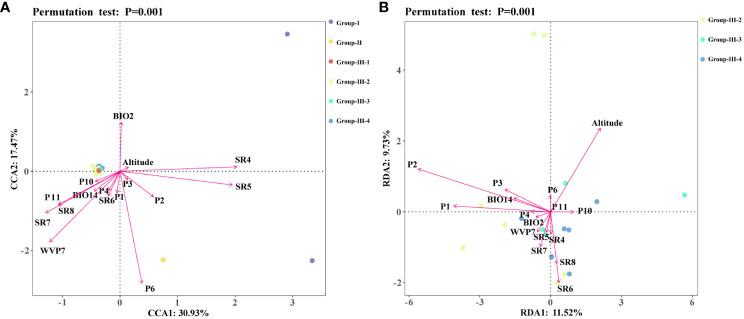
Redundancy analysis (RDA) or canonical correlation analysis (CCA) of 16 high contribution environmental variables and genetic structures. Different colored dots represented different Groups, and different arrows represented environmental variables. **(A)** The results of CCA analysis. **(B)** The results of RDA analysis.

A CCA analysis of the sample allele frequencies from the six genetic groups and the 16 environmental variables showed that variables relating to precipitation (P1, P2, P3, P6) had longer arrows that were directed towards Group II, suggesting a strong influence (explanatory value) of these environmental variables on the genetic differentiation of *C. chinensis* var. *brevisepala* from the other *Coptis* species (**Figure 5A**). In addition, the variables SR4, SR5, P2 and altitude displayed long arrows at a small angle from Group I, indicating that these had a strong influence on the genetic differentiation of *C. teeta* (**Figure 5A**). The variable P2 had a small angle with both *C*. *chinensis* var. *brevisepala* and *C*. *teeta*, and was more inclined towards the former. This was consistent with the results of the ecologically suitable analysis, which showed that P2 was an important variable shaping the distributions of all three species, especially *C*. *chinensis* var. *brevisepala* (**Figure 4D**).

The RDA results for three cultivation subgroups showed that the level of genetic variation in Group-III-4 was positively correlated with the amount of solar radiation in spring and summer (**Figure 5B**). In comparison, nearly all of the 16 environmental variables were found to influence the genetic differentiation in Group III-2 and Group 3 to different degrees. Overall, the results showed that the main environmental factors influencing the genetic variation and domestication history of *C*. *chinensis* are altitude, solar radiation and precipitation (**Figure 4E**).

## 4 DISCUSSION

### 4.1 Population genetic diversity and structure

The results revealed relatively high levels of genetic diversity in both wild and cultivated *Coptis* germplasms. In particular, the observed heterozygosity of the *C. chinensis* var. *brevisepala* population (BAHSW) was considerably lower than expected (**Table 1**). Moreover, this population had a high inbreeding coefficient (*F*_is_ = 0.366) (**Table 1**). Indeed, inbreeding could have occurred among the small natural populations of *C. chinensis* var. *brevisepala*, which have a restricted distribution in the Middle and Lower Yangtze Valley Plain. In contrast, the observed heterozygosity of the two wild *C. teeta* populations was higher than expected. Given that *C. teeta* is a species adapted to the environments of high mountains and deep valleys in the Hengduan Mountains and the Yunnan-Guizhou Plateau, geographical isolation and limited gene flow (**Figure 1**) could have accelerated the genetic differentiation among *C. teeta* individuals. This result was further supported by the high genetic differentiation (*F*_st_ = 0.257) observed between the two wild *C. teeta* populations, TYGSW and TYJPW.

Collectively, the results of the N-J tree, PCoA and STRUCTURE analyses supported the existence of independent genetic groups for *C. teeta*, *C. chinensis* var. *brevisepala* and *C. chinensis*, suggesting that substantial genetic differentiation has occurred between these *Coptis* taxa. A further delineation of the subgroups of *C. chinensis* by structure analysis yielded four subgroups. Among these, Group III-1 comprised the wild *C. chinensis* samples (CHESW) collected from Enshi, Hubei. The CHESW population germplasm may represent the early main domestication source in the authentic region of Hubei. Under the optimal number of subgroups (K = 4), cultivated *C. chinensis* were divided into three subgroups, indicating that there may be other domestication centers besides the CHESW population, and domestication in different regions may not be completed simultaneously. For example, another wild population, CNXHW, was found to be genetically similar to the cultivated *C. chinensis* samples, as evidenced by both the N-J tree and the STRUCTURE analysis. It can be speculated that the CNXHW population comprises individuals that were accidentally established in the wild from propagules of cultivated individuals domesticated in early stages rather than genuine primitive wild *C. chinensis*. Alternatively, CNXHW population may be a relatively late-stage domestication center. This research also found that the levels of genetic diversity among wild and cultivated *C. chinensis* populations did not differ significantly. This is consistent with previous claims that the genetic diversity of cultivated *C. chinensis* populations have not been eroded by domestication (Shi et al., 2008).

The cultivated *C*. *chinensis* germplasm was divided into three subgroups (Group III-2, 3 and 4), with each group showing a distinct geographical distribution. This indicated that various climatic, habitat and geomorphological conditions have influenced the genetic constituents of cultivated *C*. *chinensis* germplasms to some degree. However, that no particularly clear boundary was apparent between the clustering patterns of cultivated germplasm and different habitats suggested that long-term domestication and cultivation had led to gene transfer and germplasm exchange among groups in different regions (Wang et al., 2020; Wang et al., 2022b). For example, several cultivated populations that were geographically separated (CYMSC from Yunnan and CHJSC from Hubei; CSJYC-2 from Sichuan, CCSZC from Chongqing and CXZPC from Shaanxi) from one another showed a lower *F*_st_ value and a higher *N*_m_ value, suggesting that artificial introduction and cultivation had occurred. This was also fully consistent with the results of the analysis of their population structure at *K* = 4 (n=307).

Owing to rampant overexploitation over the long term, most wild *Coptis* populations are currently at risk of extinction. *C*. *chinensis* has been cultivated and domesticated over a long period of time through mutual introduction and commercial trade in different regions. The Mantel test showed a lack of genetic variation among populations of *C. chinensis* cultivated in different regions (**Supplementary Figure 1**). This suggests that a small genetic bottleneck event possibly occurred during the domestication of *C*. *chinensis*. This analyses further revealed that the level of genetic variation within populations was higher than that among populations, as was reported for *Angelica sinensis* (Liu et al., 2020).

### 4.2 Ecological requirements of three *Coptis* species

Previously, based on an analysis incorporating BIO1-19, Zhao et al. reported that the potentially suitable areas for *C*. *chinensis* were concentrated in the region extending from the Sichuan Basin to the middle and lower reaches of the Yangtze River (Zhao et al., 2021), and the distributions of potentially suitable areas for three *Coptis* species as well as the potential effects of climate change were also modelled in recent studies (Li et al., 2020b; Wang et al., 2022a).

In the present study, a comprehensive suite of 68 environmental variables (including BIO1-19) was used to predict the distribution of suitable areas for three *Coptis* species. Based on the present findings, the distribution of areas suitable for *C*. *chinensis* is generally consistent with those reported previously (Zhao et al., 2021). Furthermore, the results showed that highly suitable areas for the species are mainly distributed across the Sichuan Basin and in several plains located in the middle and lower reaches of the Yangtze River. The study found that there was a high degree of geographical correspondence between the three red high suitability regions and the three cultivated *C. chinensis* subpopulations, which further supported the domestication of *C. chinensis* in different suitable regions and the generation of germplasm with different genetic backgrounds through multiple domestication centers. In addition, the areas suitable for *C*. *chinensis* var. *brevisepala* are concentrated in the middle and lower reaches of the Yangtze River, while most areas suitable for *C*. *teeta* are located in Yunnan. The distinct distributions of the three *Coptis* species further support their independent genetic relationships. Therefore, based on the extensive domestication history of *Coptis*, *C*. *teeta*, which occupies a more basal position in the *Coptis* phylogeny, has had a longer population history than the other two species, and derived sister species such as *C*. *chinensis*, *C*. *omeiensis* (Chen) C. Y. Cheng and *C*. *deltoidea* as it reached the Sichuan Basin in the northeast, before deriving the variety *C*. *chinensis* var. *brevisepala* in the southeast.

According to experienced breeders, *Coptis* plants require environments with low temperatures, high levels of humidity and little solar radiation. Therefore, in this study, in addition to BIO1-19 (mainly temperature and precipitation), other environmental variables such as solar radiation, altitude, and water vapor pressure, as well as monthly data, were added as environmental factors. In particular, solar radiation and altitude have high contribution values in the ecological suitability analysis of *Coptis* plants, indicating their environmental driving role in the adaptive evolution process of *Coptis* plants to a certain extent. These previous observations are further confirmed by the findings of the present study. Separate environmental variables had different effects on the distributions of the three *Coptis* species. The environmental variables that most influenced their distributions were altitude, as well as the amount of solar radiation and precipitation, which both varied seasonally. This finding also reflected the extensive effects of environmental change on the evolution of *Coptis* species. From *C*. *teeta* to *C*. *chinensis*, solar radiation and precipitation were developed in the directions of summer to spring and spring to autumn, respectively; that is, *Coptis* species have been continuously adapting to novel seasonal changes in environmental conditions. The differences in monthly environmental variable data in the ecological suitability analysis of three species also reflected that *Coptis* plants not only adapt to changes in geographical environment, but also include seasonal changes. Such information can help guide the conservation and cultivation of different *Coptis* species.

### 4.3 Impacts of environment on genetic structure of three *Coptis* species

The results demonstrated that genetic variation, the foundation of species survival and biodiversity (Blanquart et al., 2013; Li et al., 2021), is largely influenced by changes in the environment. In the results of the CCA analysis, almost all environmental variables pointed to Groups I, II and III, which were represented by different *Coptis* species. The difference of solar radiation and precipitation caused by the environment showed key driving forces for the adaptive evolution of *Coptis* species. This was also consistent with the results of the Mantel test. Similarly, in the RDA analysis of the cultivated *C. chinensis* populations, no particularly significant correlation between the subgroups and environmental variables. However, considering that *C. chinensis* has a low domestication bottleneck and has been in introduction and cultivation for a long time, different environmental variables have shown a high driving force on the overall genetic variation of the cultivated *C. chinensis* populations. Especially the altitude, precipitation in spring, and solar radiation in autumn have formed three different directional clusters guiding the domestication direction of *C. chinensis*. In particular, the relatively concentrated Group III-4 from the high mountains around the Sichuan Basin was strongly associated with the amount of solar radiation in spring and summer. It can be inferred that the mountains around the Sichuan Basin reach high altitudes and are therefore exposed to different levels of solar radiation. This may have caused the variation in the levels of genetic diversity and in the contents of the active components of different *Coptis* accessions. Additional investigations on the metabolic components of different *Coptis* populations will further benefit efforts to identify ideal germplasms for cultivating *Coptis* species.

## 5 Conclusion

In this study, a set of highly polymorphic genomic-SSR markers was developed based on the *Coptis chinensis* genome. A genetic analysis of 345 *Coptis* accessions from 32 geographical populations based on 22 SSR markers was conducted. A high level of genetic diversity was observed in both wild and cultivated *Coptis* populations. Most wild populations showed clear levels of genetic differentiation. Additionally, there was evidence of frequent gene flow between various cultivated *C*. *chinensis* populations, with signs of multiple artificial introductions. The genetic structure of *Coptis* was found to be characterized by three major groups, while the *C*. *chinensis* germplasm could be further divided into four subgroups. The wild *C. chinensis* population in Hubei (CHESW) may represent the major domestication center of the species. The distributions of suitable areas for the three *Coptis* species were predicted, and environmental variables shaping these distributions and the genetic structures of the species were identified. *Coptis* plants have had enormous medicinal and economic value from ancient times to the present day, but currently face the problem of endangered wild resources and chaotic introduction sources. Our research will provide theoretical support for subsequent resource protection and healthy development patterns tailored to local conditions.

## Data availability statement

The original contributions presented in the study are included in the article/**Supplementary Material**. Further inquiries can be directed to the corresponding authors.

## Author contributions

YC: Formal Analysis, Investigation, Methodology, Resources, Software, Validation, Visualization, Writing – original draft, Writing – review & editing. CL: Data curation, Formal Analysis, Writing – review & editing. WL: Investigation, Supervision, Writing – review & editing. XT: Resources, Validation, Writing – review & editing. JH: Data curation, Software, Writing – review & editing. BW: Funding acquisition, Writing – review & editing. DL: Funding acquisition, Methodology, Validation, Writing – review & editing. YL: Funding acquisition, Investigation, Writing – review & editing.

## Supplementary material

Supplementary Figure 1 The mantel test of all populations and *C. chinensis* populations.
