# Supplementary figures and images for: Population genetic variation and geographic distribution of suitable areas of Coptis species in China
Source: Front Plant Sci. 2024 Mar 19;15:1341996. doi: 10.3389/fpls.2024.1341996 (PMC10985201; doi:10.3389/fpls.2024.1341996)

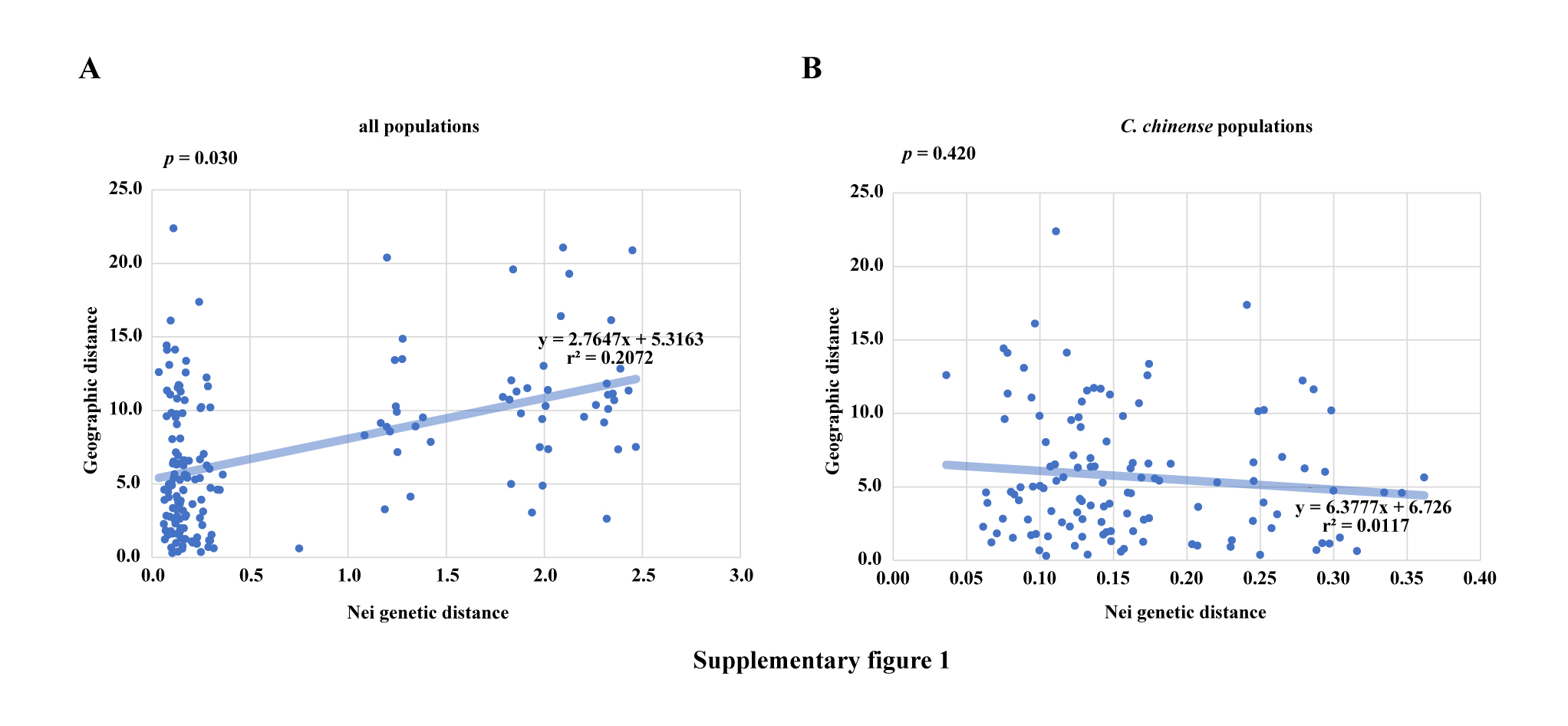

Supplement: Supplementary Figure 1 — The mantel test of all populations and C. chinensis populations. [file Image_1.jpeg]
